# Cryo-EM fibril structures from systemic AA amyloidosis reveal the species complementarity of pathological amyloids

**DOI:** 10.1038/s41467-019-09033-z

**Published:** 2019-03-07

**Authors:** Falk Liberta, Sarah Loerch, Matthies Rennegarbe, Angelika Schierhorn, Per Westermark, Gunilla T. Westermark, Bouke P. C. Hazenberg, Nikolaus Grigorieff, Marcus Fändrich, Matthias Schmidt

**Affiliations:** 1 Institute of Protein Biochemistry, Ulm University, 89081 Ulm, Germany; 2 Janelia Research Campus, Howard Hughes Medical Institute, Ashburn, VA 20147 USA; 3 Institute of Biochemistry and Biotechnology, Martin-Luther-University, 06120 Halle (Saale), Germany; 4 Department of Immunology, Genetics, and Pathology, Uppsala University, Uppsala, SE-751 85 Sweden; 5 Department of Medical Cell Biology, Uppsala University, SE-75123 Uppsala, Sweden; 6 Department of Rheumatology & Clinical Immunology, University of Groningen, University Medical Center Groningen, 9700 RB Groningen, The Netherlands

## Abstract

Systemic AA amyloidosis is a worldwide occurring protein misfolding disease of humans and animals. It arises from the formation of amyloid fibrils from the acute phase protein serum amyloid A. Here, we report the purification and electron cryo-microscopy analysis of amyloid fibrils from a mouse and a human patient with systemic AA amyloidosis. The obtained resolutions are 3.0 Å and 2.7 Å for the murine and human fibril, respectively. The two fibrils differ in fundamental properties, such as presence of right-hand or left-hand twisted cross-β sheets and overall fold of the fibril proteins. Yet, both proteins adopt highly similar β-arch conformations within the N-terminal ~21 residues. Our data demonstrate the importance of the fibril protein N-terminus for the stability of the analyzed amyloid fibril morphologies and suggest strategies of combating this disease by interfering with specific fibril polymorphs.

## Abstract (web summary)

Systemic AA amyloidosis is caused by misfolding of the acute phase protein serum amyloid A1. Here the authors present the cryo-EM structures of murine and human AA amyloid fibrils that were isolated from tissue samples and describe how the fibrils differ in their fundamental structural properties.

## Introduction

The formation of amyloid fibrils represents the unifying feature of a range of debilitating human disorders from neurodegenerative Alzheimer’s and Parkinson’s diseases to the various forms of systemic amyloidosis^1,2^. Systemic AA amyloidosis represents one of the most abundant forms of systemic amyloidosis that affects humans and over 50 animal species (mammals and birds)^3,4^. The disease arises in mice and humans from the misfolding of the acute phase protein serum amyloid A1 (SAA1)^1,3,5^. Strong inflammatory stimuli drastically increase the serum levels of this protein, reaching peak concentrations of more than 1 mg/mL^3,5,6^. High SAA1 levels are the prerequisites for developing the disease in humans, which typically follows chronic inflammatory conditions, such as tuberculosis, leprosy, rheumatoid arthritis, and familial Mediterranean fever^3,6^. Current treatment standards aim to reduce the serum SAA1 levels but are unable to control the disease in all cases^3,5^. Amyloid-specific therapies are not available.

AA amyloid fibrils are characterized by a linear morphology and a cross-β structure^7,8^. They are polymorphic and multiple fibril morphologies can be found, when extracting AA amyloid fibrils from diseased tissue^9,10^. Amyloid fibrils underlie central aspects of the pathology of systemic amyloidosis as they form massively sized deposits that physically impair and distort the affected tissues^6,11^. In AA amyloidosis, amyloid is typically found in spleen, liver, and kidneys^3,6^, but in particular renal AA amyloid is a health burden and leads to proteinuria if not to end-stage kidney disease or death^3,6^. Oligomeric fibrillation intermediates exacerbate the pathogenic effects of AA amyloid fibrils, similar to their toxicity in other amyloid diseases^2,3,5^.

Amyloid fibrils also underlie the prion-like characteristics of systemic AA amyloidosis in mice and several other animal species^3,4,12,13^. Injection of purified amyloid fibrils, fibril fragments, oligomers or spleen extracts from amyloidotic donors into inflamed mice transmits the disease between animals^12,14^. Transmission is possible via oral uptake and across different species^4,12^. For example, feeding of inflamed mice with AA containing foie gras from goose^15^ or injection of human spleen extracts or purified human AA amyloid fibrils provokes disease in the recipient^4,16^. However, the efficiency by which other murine AA amyloid fibrils induce murine AA amyloidosis is higher than that of amyloid fibrils from other species, including humans^4,16^, resembling the species barrier as in the transmissible spongiform encephalopathy (TSE)^17^.

Despite considerable data demonstrating the pathogenic relevance of AA amyloid fibrils, little is known about their atomic structures. To investigate the molecular basis of the systemic AA amyloidosis, we here use electron cryo-microscopy (cryo-EM) and determined the structures of AA amyloid fibrils from a patient and from a diseased mouse. The observed structures provide insight into the mechanism of misfolding and fibril cross-seeding, and they suggest possibilities of interfering with the amyloid fibril formation as it occurs in disease.

## Results

### Primary structure of the human and murine fibril proteins

AA amyloid fibrils were extracted from the kidney of an AA amyloidotic patient and from the spleen of a mouse diseased with systemic AA amyloidosis. The used extraction procedure was previously established to avoid harsh physical or chemically denaturing conditions and to maintain the fibril morphology^9,10^. Transmission electron microscopy (TEM) shows more than 90% of the murine and more than 98% of the human fibrils to belong to a dominant fibril morphology (Supplementary Figure 1). The dominant morphology of the mouse shows a width of 11.8 ± 0.5 nm and a cross-over distance of 75.7 ± 1.3 nm. The dominant human morphology is 8.1 ± 0.5 nm wide and possesses a cross-over distance of 55.2 ± 1.7 nm (measurements were obtained from cryo-EM images). Denaturing gel electrophoresis confirms the purity of the fibril extracts (Supplementary Figures 2a, 2c) with fibril proteins migrating at ~6 kD (mouse) and ~4 kDa (human). Corresponding to previous observations^12^, mass spectrometry (MS) shows that the mouse fibril proteins are N-terminal fragments of murine mSAA1.1 protein (Supplementary Figure 2b) with mSAA1.1(1–76), mSAA1.1(1–82), and mSAA1.1(1–83) being particularly abundant (Supplementary Table 1). The human fibril proteins originate from human hSAA1.1 and mainly represent the fragments hSAA1.1(2–64) and hSAA1.1(2–47) (Supplementary Figure 2d and Supplementary Table 1). MS did not reveal any posttranslational modifications of the amino acid side chains.

### Global topology and β-sheet twist of the two fibrils

**Fig. 1:**
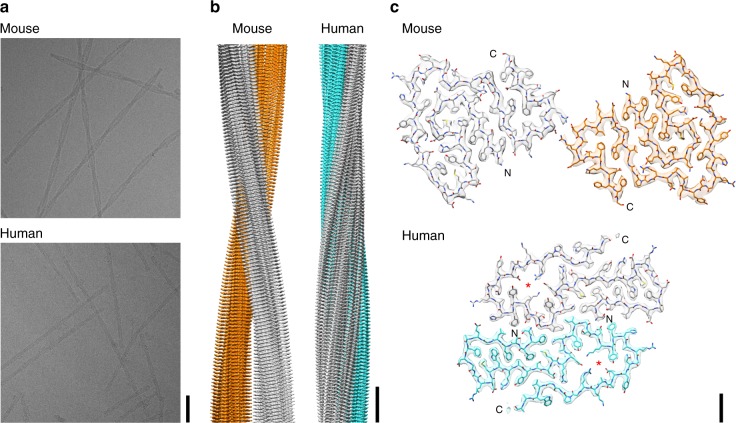
Cryo-EM reconstructions of a murine and human AA amyloid fibril. **a** Cryo-EM images (scale bar: 50 nm). **b** Side views of the reconstructions. The two protein stacks of a fibril are colored gray and cyan (human) or gray and orange (murine) (scale bar: 50 Å). **c** Cross-sectional view of one molecular layer. The densities are superimposed with the molecular models. Red asterisks indicate the cavities (scale bar: 10 Å)

Using cryo-EM, we determined the three-dimensional (3D) structure of the mouse fibril at a resolution of 3.0 Å and of the human fibril at a resolution of 2.7 Å (Fig. 1, Supplementary Figure 3 and Supplementary Table 2). The 3D maps show well-resolved densities from the amino acid side-chains and allowed us to trace the polypeptide chain within the density. Two-dimensional (2D) projections of the reconstructed densities and the fitted models correlate well with the respective 2D class averages (Supplementary Figure 4). The ordered part of the density of the mouse fibril corresponds to residues 1–69 of mSAA1.1, the ordered part of the human fibril to hSAA1.1 residues 2–55. Adjacent to the C-terminal ends of their ordered parts, both fibril reconstructions show diffuse density (Supplementary Figure 5), indicating that C-terminal tails of the fibril proteins, corresponding to residues 70–83 in the murine and 56–67 in the human fibril proteins, are structurally disordered. Both fibrils are polar and possess a pseudo-2_1_ symmetry, resembling several other cross-β fibrils^18–20^. They are double helical structures consisting of two stacks of protein molecules that are oriented in parallel to one another but show an offset of half a cross-β repeat, consistent with their pseudo-2_1_ symmetry (Fig. 1b and Supplementary Figure 4).

**Fig. 2:**
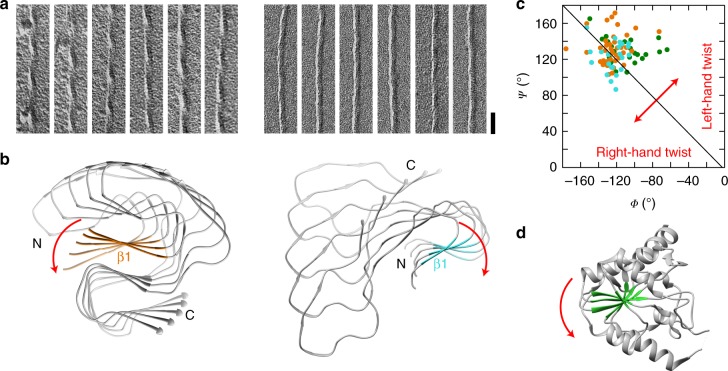
Different handedness of the β-sheet twist in the murine and the human fibril. **a** TEM images of murine (left) and human (right) AA amyloid fibrils after platinum side shadowing (scale bar: 50 nm). **b** Left-hand β-sheet twist of the murine fibril illustrated for sheet β1 (orange) and right-hand twist of the β-sheet of the human fibril illustrated for β2 (cyan). Every tenth molecule along the fibril axis displayed. **c** Ramachandran plot of all residues within the β-strands of human (cyan), murine fibril (orange) and in the globular protein of phosphoglycerate kinase (green). **d** Ribbon diagram illustrating the left-hand β-sheet twist of human phosphoglycerate kinase (PDB 3C39, residues 1–202)

Platinum side shadowing and TEM demonstrate the left-hand twist of the mouse fibril and its underlying cross-β sheets (Fig. 2a, b). A left-hand twist is defined here by viewing along the backbone hydrogen bonds or along the main fibril axis, and it is the canonical direction of the β-sheet twist in globular proteins^21^ and the predominant twist observed in cross-β fibrils^18–20,22^. By contrast, platinum side shadowing demonstrates that the human fibril is right-hand twisted and contains right-hand twisted β-sheets (Fig. 2a, b). The fibril twist arises from the distribution of the backbone *Φ*/*Ψ* dihedral angles within the Ramachandran plot^21^ such that *Φ*/*Ψ* pairs occurring to the left of the −*Φ* = *Ψ* diagonal of the plot indicate a right-hand twist and vice versa.

Both amyloid fibrils show a distribution of the β-sheet *Φ*/*Ψ* dihedral angles that is close to the diagonal (Fig. 2c), indicating a low β-sheet twist. The sum *Ψ* + *Φ* yields a value of −2 ± 20° for the β-sheet residues of the human fibril and of 8 ± 20° for the murine fibril, indicating a slight preference for a right-hand twist in the human and for a slight left-hand twist in the murine fibril. By comparison, the β-sheets in a natively folded domain of phosphoglycerate kinase yields a much higher value of 20 ± 23°, indicating a strongly left-hand twisted β-sheet (Fig. 2c, d). While a right-hand twist has previously been associated with a sample of amyloid-like fibrils from hSAA1.1(1–12) peptide^23^, our demonstration of a right-hand twisted fibril in the human AA amyloidosis shows that non-canonically twisted β-sheets can exist in nature in the context of pathological aggregates.

### Structural compactness and fold of the fibril proteins

**Fig. 3:**
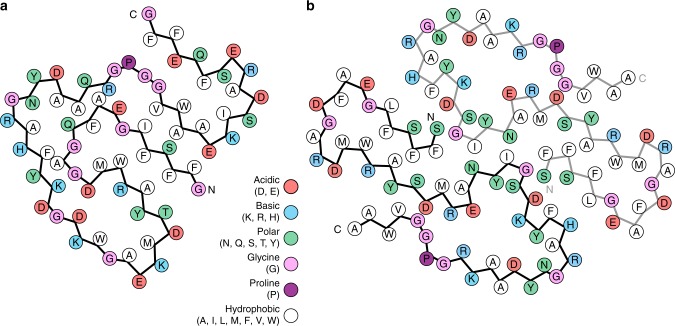
Schematic view of the fibril protein illustrating the complementary packing. **a** Murine fibril protein. **b** Molecular layer of the human fibril, consisting of two fibril proteins

**Fig. 4:**
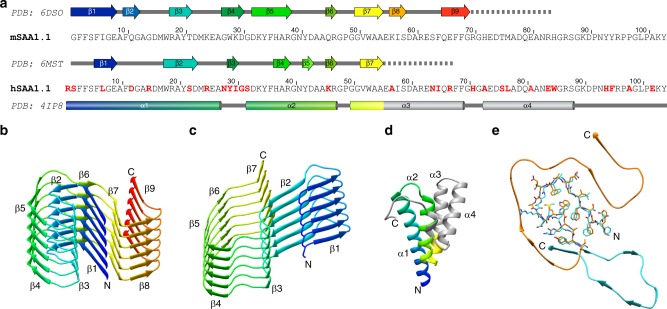
Secondary structure of the fibril proteins. **a** Sequence comparison of mSAA1.1 and hSAA1.1. Red: amino acid substitutions in hSAA1.1 compared to mSAA1.1. Secondary structural assignments according to the respective PDB entries. Cylinders: α-helices; arrows: β-strands; dotted line: segment of the fibril protein not resolved by cryo-EM. **b**, **c** Six molecular layers of one protein stack of the murine (**b**) and human fibril (**c**). **d** Crystal structure of hSAA1.1 (PDB 4IP8)^24^. Residues 1–55 are rainbow-colored from N (blue) to C (red). The colors of panels **c** and **d** are corresponding. Gray: C-terminal residues disordered or missing in the human fibril protein. **e** Superimposition of residues 1–21 of the murine fibril protein and residues 2–22 of the human fibril protein, showing the close similarity of the β-arch fold

The fold of the murine protein is compact and shows a high degree of complementarity (Figs. 1c, 3a). It lacks large, water-filled cavities and differs in this property from the human fibril protein. The human fibril encloses a clearly discernible cavity that contains many charged and polar amino acid residues (Figs. 1c, 3b), making it likely that the vacant space in the cavity is filled with water. Both fibril proteins adopt all-beta folds (Fig. 4a–c) and differ sharply from the known globular conformations of SAA family members^24,25^. Globular hSAA1.1 possesses an all-alpha protein fold and lacks all elements of β-sheet structure (Fig. 4d). The human fibril protein contains seven β-strands (β1–β7), the murine fibril protein nine (β1–β9) (Fig. 4a–c).

Our data are consistent with previous observations of β-sheet structure in AA amyloid fibrils^7,11^ and demonstrate an orientation of the backbone hydrogen bond donor and acceptor groups that is largely perpendicular to the main fibril axis. The β-strands are roughly oriented cross to this direction, although they are slightly tilted with respect to the main fibril axis (Fig. 1b), reminiscent of recent cryo-EM structures of cross-β fibrils^18–20^. The murine and the human fibril possess in-register, parallel cross-β sheets (Fig. 4b, c) and adopt highly similar β-arch conformations at the protein N-termini. In particular residues 4–22 of the human fibril are closely related in structure to the homologous residues 3–21 of the murine fibril (Fig. 4e). This segment encloses in both fibrils a densely packed hydrophobic core that is formed by residues Phe3, Phe5, Phe10, Met16, Trp17 and Ala19 of mSAA1.1 and the homologous residues in the human fibril (Figs. 1c, 3).

The more C-terminal segments differ substantially in conformation between the human and the murine fibril (Fig. 4e). In the murine fibril they wrap around the N-terminal β-arch such that the resulting protein fold superficially resembles the Greek key topology, similar to α-synuclein fibrils^20^. However, the fibrils lack the intramolecular backbone hydrogen bonds between the β-strands that are required to form a Greek key, and the intramolecular strand-strand interactions of the fibril protein are instead formed by the amino acid side-chains. We refer to this motif as an ‘amyloid key’. The human fibril protein contains no amyloid key fold.

### Cross-sectional interactions stabilizing the fibrils

**Fig. 5:**
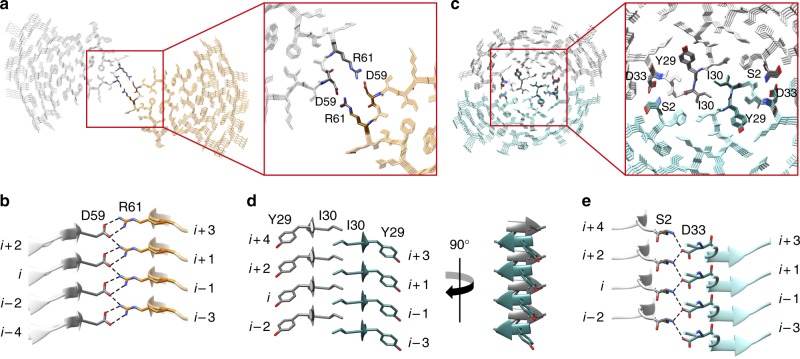
Interactions between the two protein stacks in the murine and human fibril. **a** Cross-sectional view of four molecular layers of the murine fibril with a close-up on the stack-stack interface. **b** Side view showing the ladder of ionic residues which make bidentate interactions between the two murine protein stacks. **c** Cross-sectional view of four molecular layers of the human fibril with a close-up on the interface between the protein stacks. **d** Cross-stack steric zipper formed by β-sheet β3 (residues Tyr29 and Ile 30) of the human fibril. **e** Cross-stack interactions between the α-amino group of Ser2 and the side chain carboxyl group of Asp33 from the other protein stack

Another substantial difference between the human and the murine fibril protein concerns the packing interface between the two protein stacks. This interface is extremely small in the murine fibril. Two residues (Asp59 and Arg61) make bidentate, reciprocal salt bridges with the respective residues in the opposing protein stack (Fig. 5a, b). The human fibril exhibits a considerably larger interface containing polar, ionic, and hydrophobic cross-stack interactions. The center of the human fibril is formed by a steric zipper, which shows a self-complementary packing of the sheets β3 from the two protein stacks (Fig. 5c, d). The sheet β3 consists of only two residues (Tyr29 and Ile30) and arises from a sequence segment that shows the largest difference between the murine and the human fibril protein (Fig. 4a). Adjacent to the zipper we find buried salt bridges between the N-terminal α-amino groups and the β-carboxyl groups of Asp33 from the other protein stack (Fig. 5e).

**Fig. 6:**
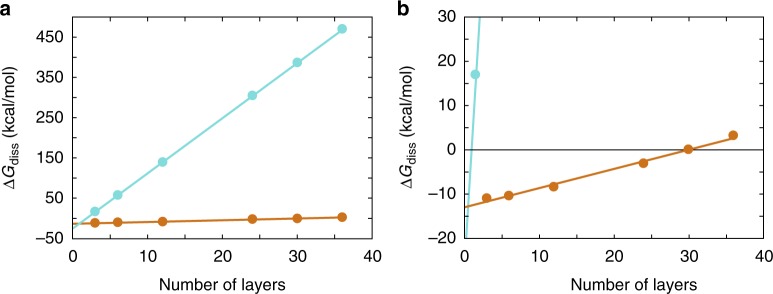
Estimation of the value of Δ*G*_diss_ for the human and murine fibril. Dependence of the stack-stack interactions of the murine (orange) and human fibril (cyan) on the number of molecular layers as estimated by the program PDBePISA^56^. The panel **b** is a close-up of the left panel **a** to show the intersection of the Δ*G*_diss_ values with Δ*G*_diss_ = 0

The much more extensive packing interface of the human fibril resulted in much higher Gibbs free energy that is required to dissociate the two protein stacks (Δ*G*_diss_) as estimated with the program PDBePISA (Fig. 6). For the human fibril we obtain an average Δ*G*_diss_ value of 13.7 kJ/mol for the cross-stack interactions per molecular layer. For the murine fibril this value is only 0.4 kJ/mol, which demonstrates that the cross-stack interactions are much weaker in this fibril morphology. Related to the stronger cross-stack interactions of the human fibril, we find Δ*G*_diss_ to become positive (and the cross-stack interactions to be stabilizing) if a fibril fragment consists of two or more molecular layers. By contrast, at least 30 molecular layers are required for the murine fibril to form a stable stack-stack interface (Fig. 6b).

The water exposed surfaces of both fibrils are rich in hydrophilic amino acid residues, while the fibril cores show complex patterns of ionic, polar, and hydrophobic interactions. These patterns arise from the complementarity of the structural elements forming the cores of the human and the murine fibril (Fig. 3). Particularly remarkable is the mutual charge compensation of six buried ionic residues (Asp15, Arg18, Asp30, Lys33, Glu8, and Arg46) that form a network of salt bridges in the murine fibril (Fig. 3a). The human fibril also shows six internal ionic residues (Asp22, Arg24, Glu25, Lys33, Asp42, and Arg46) that participate in defining the cavity wall (Fig. 3b).

### Protein interactions in direction of the fibril main axis

**Fig. 7:**
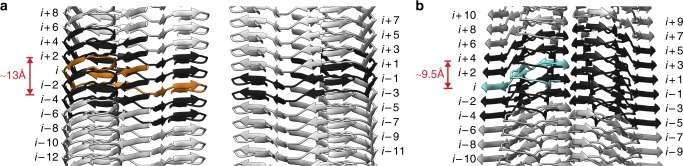
Axial rise of the fibril protein interdigitates the structure. **a** Side view of the murine fibril. The murine fibrils shows a 13 Å chain rise between the carbonyl carbons of Glu25 and Val51. Molecule *i* (orange) interacts with six other molecules (black). **b** Side view of the human fibril. The human fibril shows a 9.5 Å chain rise between the carbonyl carbons of Phe36 and Pro49. Molecule *i* (cyan) interacts with ten other molecules (black)

Based on our structure we can identify several types of interactions that stabilize the fibrils in the direction of the fibril main axis (Supplementary Figure 3b). These are the backbone hydrogen bonds of the cross-β sheets and an in-register stacking of the amino acid residues, in particular of polar or aromatic side chains (Supplementary Figure 3b), along the fibril axis. The pseudo-2_1_ symmetry (Fig. 1b and Supplementary Figure 4) and the staggering of the two protein stacks along the fibril axis produce interactions between chain *i* of one stack and two chains (*i* + 1 and *i *− 1) in the other stack. Examples thereof are the steric zipper of the human fibril (Fig. 5c, d) and the cross-stack salt bridges of the murine fibril (Fig. 5b). The fibril proteins are also not entirely flat and show a height change in the direction of the fibril axis (~13 Å and ~9.5 Å for the murine and human protein, respectively, Fig. 7). Resulting from this height change, each murine fibril protein interacts with six other protein molecules, four within the same stack and two of the opposite stack (Fig. 7a). Each human fibril protein interacts with ten other protein molecules (Fig. 7b). The non-planarity of the murine fibril protein originates from the tilt of the protein stacks with respect to the fibril axis (Fig. 1b) and a GPGG motif (residues 47–50) that induces a ~5.5 Å height change of the polypeptide chain relative to the fibril axis.

The height change could lead to different mechanisms or different kinetics of fibril outgrowth at the two fibril ends^19^ and is important for the formation of several intermolecular interactions that sterically interdigitate the fibril structures along their main axes. For example, there is an intermolecular packing between strand β7 from molecule *i* of the mouse fibril and strands β1 from molecules *i* − 2 and *i* – 4 in the same protein stack (Supplementary Figures 6a, 6c). Moreover, many of the aforementioned buried interactions, including the buried networks of salt bridges described above in the murine fibril, run across different molecular layers (Supplementary Figures 6d, 6e).

## Discussion

We here present the molecular structures of two fibrils from systemic AA amyloidosis. Both analyzed filaments show an arrangement in which the protein molecules are stacked up in the direction of the fibril axis. They form intermolecular β-sheets that are connected by hydrogen bonds running in the direction of the fibril axis (Supplementary Figure 3b). The observed strand-strand distance of ~4.8 Å explains the previously reported 4.76 Å X-ray spacing^7^ and provides molecular views of the cross-β structure, the generic structural element of amyloid fibrils^26,27^. Our structures differ in several respects from traditional representations of amyloid fibrils in systemic amyloidosis^27^, as it involves parallel β-sheets (Fig. 4b, c) and β-strands that are not fully perpendicular to the fibril axis but slightly tilted (Fig. 1b). We further find a height change of the protein molecules (Fig. 7) and a β-sheet twist that can be right-hand (Fig. 2a, b), differing sharply from canonical β-sheet twist in globular proteins^21^ and most previously analyzed cross-β fibrils^18–20,22^.

Our cryo-EM reconstructions reveal several stabilizing structural elements, such as alternating patterns of buried ionic, polar, and hydrophobic interactions (Supplementary Figure 6), a steric zipper (in the human fibril) (Fig. 5c, d) and the interdigitation of the fibril proteins along the main fibril axis (Fig. 7). These structural elements provide a basis for the stiffness and mechanical resistance of these fibrils and thus the central aspects of the pathology of systemic amyloidosis^11^. The highly compact fold of the fibril proteins that is reminiscent of the packing within a core of a well-folded globular protein is remarkable given that the amino acid sequence of the precursor proteins have been optimized by nature to provide complementarity and structural compactness within a radically different and mainly α-helical conformation (Fig. 4d). However, only the N-terminal parts of the two SAA1 proteins are able to adopt this compact structure in the fibril state, while the more C-terminal segments are structurally disordered or cleaved off (Supplementary Figures 2, 5). The α-to-β transition documented here for systemic AA amyloidosis provides an analogy to the well-known α-to-β transition of the prion protein in TSE^17^ and implies that the native structure must be at least partially unfolded in order to allow the chains to self-assemble into a cross-β fibril.

The highly similar folds of murine and human SAA proteins in available crystal structures^24,25^ are in contrast to the clear differences between the conformations of the two fibril proteins (Fig. 4e). Comparison of the sequences of hSAA1.1 or hSAA1.3 with the fold of the murine protein (and vice versa) demonstrates that these sequences are incompatible with the fibril fold adopted by the other species (Supplementary Figure 7a–c). Yet, there are clear similarities at the first ~21 residues that adopt a β-arch fold in both species. The partial complementarity of the murine and human fibril protein folds imply a resistance of the murine protein to adapt to the human fibril fold (except at the N-terminal ~21 residues), which is in accordance with observations of a limited cross-seeding efficiency of human amyloid fibrils or tissue extracts in mice^4,16^.

Our data identify the N-terminal ~21 residues of the fibril proteins, the most hydrophobic and amyloidogenic segment of the protein sequence and a driver of systemic AA amyloidosis^28–30^, as crucial for structuring disease-associated AA amyloid fibrils. That is, single amino acid changes within this region can make the protein incompatible with the observed fibril architecture. For example, hSAA1.1 contains an additional N-terminal Arg residue compared to mSAA1.1 (Fig. 4a). Interestingly, however, this residue is missing in our human fibril protein (Supplementary Figure 2 Supplementary Table 1) and incompatible with the packing of the observed fibril structure (Fig. 5c). Our reconstruction shows that the N-terminus of the human protein, which lacks the N-terminal Arg, is located within the tightly packed fibril core (Fig. 5c). The N-terminal α-amino groups form salt bridges to the β-carboxyl groups of Asp33 (Fig. 5e). As a significant fraction of the hSAA1.1 that is circulating in the blood in the course of an acute phase response lacks the N-terminal Arg residue^31^, our structure suggests that it is this fraction of the protein that constitutes the precursor of the fibril. However, arginated hSAA1.1 forms fibrils in vitro^32^ and may also do so in certain AA patients^33^, suggesting that the presence of an N-terminal Arg does not generally block the assembly of hSAA1.1 into cross-β fibrils but that it is specifically incompatible with the fibril morphology described here. Our structures are representative for the samples, due to the strong predominance of the main morphologies. Compared to systemic AA amyloidosis in other species, such as island fox and domestic goat^9^, the degree of polymorphism is less profound, but nevertheless present.

Another example for the destabilizing effect of changes at the protein N-terminus is provided by the fact that CE/J mice and *Mus musculus czech* are unable to develop AA amyloidosis due to the expression of the variant SAA proteins mSAA2.2 and mSAA1.5, respectively^34,35^. While mSAA1.5 has hardly been investigated, mSAA2.2 forms amyloid-like fibrils in vitro^32^, similar to pathogenic mSAA1.1^30,32^, demonstrating the resistance of CE/J mice towards development of systemic amyloidosis cannot be explained with an inability of mSAA2.2 protein to convert into cross-β fibrils. Based on our structure, however, we find that the sequences of both proteins, mSAA2.2 and mSAA1.5, are incompatible with the specific packing of the analyzed fibril structure. mSAA2.2 differs at six positions from mSAA1.1, three of which (Ile6Val, Gly7His, Ala101Glu) are also present in mSAA1.5 (Supplementary Figure 7a). Two of these changes are unable to explain pathogenicity. Residue 101 is absent in the fibril protein (Supplementary Figure 2b) and the Ala101Glu mutation does not prevent amyloidosis as SJL/J mice^36^ express the Ala101Glu mutation in mSAA1.5 (Supplementary Figure 7a). Ile6Val is synonymous and consistent with the observed fibril morphology. By contrast, the Gly7His mutation places a bulky, charged residue into the tightly packed, hydrophobic core of the N-terminal β-arch (Supplementary Figure 7d) and is thus disruptive to the central structural element of the fibril. The importance of residue 7 for fibril formation is further corroborated by the fact that human pathogenic hSAA1.1 and hSAA1.3 possess a Gly residue at this site, while mSAA2.1, mSAA3, and mSAA4, which are unable to form amyloid in vivo^37^, contain a bulky, positively charged residue at position 7 (Supplementary Figure 7a).

These observations imply that the presence of charged residues at strategic sites prevents the formation of specific, disease-associated fibril morphologies rather than preventing any form of cross-β assembly. These findings suggest two possibilities of fibril morphology-specific strategies to combat the development of amyloid diseases. The first possibility arises from our observations made with the human fibril and its incompatibility with hSAA1.1 containing an N-terminal Arg. Hence, preventing the removal of the N-terminal Arg should prevent the formation of the observed fibril structures and thus the development of amyloidosis, at least within certain groups of individuals. The second possibility arises from the ability of mSAA1.5 and mSAA2.2 to render *M. musculus czech* and CE/J mice resistant to development of systemic AA amyloidosis. Both proteins confer this resistance also to animals that expresses the variant protein in addition to the pathogenic mSAA1.1 protein^34,35^. That is, the variant proteins are able to prevent the formation of fibrils from normally pathogenic mSAA1.1 protein and thus are able to act as an inhibitor of amyloid fibril formation in vivo.

## Methods

### Source of the murine AA amyloid fibrils

Fibrils were purified from AA amyloidotic mice. Female 6- to 8-week-old NMRI mice (Charles River Laboratories) received on day 0 a single 0.1 mL injection of a 0.1 mg/mL protein solution containing murine AA amyloid fibrils into the lateral tail vein. Immediately afterwards, the animals received a subcutaneous injection of 0.2 mL freshly prepared 1 % (w/v) solution of AgNO_3_ in distilled water. The AgNO_3_ injection was repeated using 0.1 mL after 7 and 14 days. Animals were euthanized with CO_2_ on day 16 and spleens were removed subsequently. AA fibrils from amyloid-laden mouse spleen were extracted based on a preexisting protocol^10^. In brief, 100 mg of tissue material were washed five times with 1 mL Tris calcium buffer (20 mM Tris, 138 mM NaCl, 2 mM CaCl_2_, 0.1% (w/v) NaN_3_, pH 8.0). Samples were centrifuged at 3100 × *g* for 1 min at 4 °C. The pellet was resuspended in 1 mL of 5 mg/mL Clostridium histolyticum collagenase (Sigma) in Tris calcium buffer. After incubation overnight at 37 °C (horizontal shaking at 750 rpm) the tissue material was centrifuged at 3100 × *g* for 30 min at 4 °C. The pellet was resuspended in 1 mL Tris ethylenediaminetetraacetic acid (EDTA) buffer (20 mM Tris, 140 mM NaCl, 10 mM EDTA, 0.1% (w/v) NaN_3_, pH 8.0) and homogenized. The homogenate was centrifuged for 5 min at 3100 × *g* at 4 °C. This step was repeated two times. Afterwards, the tissue pellet was homogenized in 200 µL ice cold water. The homogenate was centrifuged for 5 min at 3100 × *g* at 4 °C and the fibril containing supernatant was stored. This step was repeated four times. All animal experiments were approved by the Regierungspräsidium Tübingen.

### Source of the human AA amyloid fibrils

A 48-year-old woman was diagnosed with chronic pulmonary obstructive disease characterized by recurrent bronchial infections. Eight years later she was diagnosed with progressively erosive seropositive rheumatoid arthritis. Amyloid was detected in a rectum biopsy. At the age of 69 the proteinuria and loss of renal function had progressed and a kidney biopsy showed AA amyloid by Congo red birefringence and immunohistochemistry. After a pneumonia the clinical situation deteriorated rapidly and she died. Informed consent was obtained from the family for autopsy and analysis of the amyloid deposits. The autopsy showed renal vein thrombosis and extensive AA amyloidosis in the arteries of all organs. Thyroid, adrenal glands, spleen, and kidneys showed prominent deposition of amyloid (in all glomeruli and in the vascular walls). AA fibrils from the diseased kidney were extracted as described above. The analysis of the fibrils was performed by ethical approval of the ethical committee from Ulm University.

### Denaturing gel electrophoresis

A solution of fibrils was mixed at 3:1 ratio with 4X lithium dodecyl sulfate sample buffer (Thermo Fisher Scientific) and heated at 95 °C for 10 min. Proteins were separated on a NuPAGE 4–12 % Bis-Tris gradient gel (Thermo Fisher Scientific) using NuPAGE MES LDS running buffer (Thermo Fisher Scientific). The gel was stained for 1 h with a solution of 2.5 g/L Coomassie brilliant blue R250 in 20% (v/v) ethanol and 10% (v/v) acetic acid. The gel was destained in 30% (v/v) ethanol and 10% (v/v) acetic acid.

### Mass spectrometry

A sample of murine AA amyloid fibrils was dried by using a Vacuum Concentrator 5301 (Eppendorf) and resuspended in an equivalent volume of 6 M guanidine hydrochloride in 10 mM Tris buffer pH 8. The sample was desalted using a ZipTip (Merck Millipore). Matrix-assisted laser desorption/ionization MS spectra were recorded using an Ultraflex-II MALDI TOF/TOF mass spectrometer (Bruker) operated with Flex Control 3.0 software and externally calibrated with a protein calibration mixture (Bruker). One microliter of 2,5-dihydroxybenzoic acid solution (7 mg solved in 100 μL methanol, Bruker) was mixed with 1 μL protein solution. One microlitre of this mixture was deposited onto a stainless steel target. Based on our set up a maximum error of 2 Da was assumed.

A sample of human AA amyloid fibrils was denatured in 6 M guanidine hydrochloride, 20 mM NaPO_4_, pH 6.5 and incubated overnight at room temperature under constant agitation at 200 rpm using a circular shaker (IKA MTS2/4 digital). Afterwards, the sample was applied onto a Source 15RPC reverse-phase 3 mL column, equilibrated in 0.1% (v/v) trifluoracetic acid in water. Proteins were eluted by a linear gradient from 0 to 100% of 86% (v/v) acetonitrile, 0.1% (v/v) trifluoracetic acid solution over 35 column volumes. For electrospray, the samples were separated on a nanoAcquity UPLC, being trapped on an Waters Acquity M-Class BEH C4 300 µm × 50 mm column (5 µm particle size and 300 Å pore size), and analyzed on an Acquity M-Class BEH C4 100 µm × 100 mm analytical column (1.7 µm particle size and 300 Å pore size), at 600 nl/min over a gradient of 3% acetonitrile/0.1% formic acid to 95% acetonitrile/0.1% formic acid (v/v). The intact protein species were analyzed on a Waters Synapt G2 HDMS in time of flight MS positive mode scanning an MS range of 2000–10,000 *m/z* over a four second cycle. Based on our set up a maximum error of 2 Da was assumed.

### Negative-stain TEM

Negative-stain TEM specimens were prepared by loading 5 µL of the sample (0.2 mg/mL) onto a formvar and carbon coated 200 mesh copper grid (Plano). After incubation of the sample for 1 min at room temperature, the excess solvent was removed with filter paper. The grid was washed three times with water and stained three times with 2% (w/v) uranyl acetate solution. Grids were examined in a JEM-1400 transmission electron microscope (JEOL) that was operated at 120 kV.

### Platinum shadowing

Formvar and carbon coated 200 mesh copper grids (Plano) were glow-discharged using a PELCO easiGlow glow discharge cleaning system (TED PELLA). A 5 µl droplet of the AA amyloid fibril sample (0.2 mg/mL) were placed onto a grid and incubated for 30 s at room temperature. Excessive solution was removed with filter paper (Whatman). Grids were washed three times with water and dried at room temperature for 30 min. Platinum was evaporated at an angle of 30° using a Balzers TKR 010 to form a 1 nm thick layer on the sample. Grids were examined in a JEM-1400 transmission electron microscope (JEOL), operated at 120 kV.

### Morphological analysis

Morphological counts were obtained by visual inspection of negative-stain TEM and cryo-TEM images. Measurements of fibril width and crossover distance of 100 human and mouse fibrils each, were carried out with Fiji^38^ using cryo-TEM images. Errors represent standard deviations.

### Cryo-EM

A 4 µL (murine AA fibrils) or 3.5 µL (human AA fibrils) aliquot (0.2 mg/mL) was applied to glow-discharged holey carbon coated grids (200 mesh C-flat 2/1 for murine AA fibrils, and 400 mesh C-flat 1.2/1.3 for human AA fibrils), blotted with filter paper and plunge-frozen in liquid ethane using a Vitrobot Mark 3 (Thermo Fisher Scientific). Grids were screened using a JEM-2100 transmission electron microscope (Jeol) at 200 kV. Images were acquired using a K2-Summit detector (Gatan) in counting mode (super-resolution, murine AA fibrils) on a Titan Krios transmission electron microscope (Thermo Fisher Scientific) at 300 kV. Data acquisition parameters are listed in Supplementary Table 2.

### Helical reconstruction

Super-resolution movie frames were corrected for gain reference using IMOD^39^. Motion correction, dose-weighting and binning by a factor of 2 was done using MOTIONCOR2^40^. The contrast transfer function was estimated from the motion-corrected images using Gctf^41^. Helical reconstruction was performed using RELION 2.1^42^. Fibrils were selected manually from the aligned micrographs. Segments were extracted using a box size of ~280 Å and an inter-box distance of ~10% of the box length. Reference-free 2D classification with a regularization value of *T* = 2 was used to select class averages showing the helical repeat along the fibril axis. Initial 3D models for both fibrils were generated de novo from a small subset (200 particles per class) of the selected class averages using the Stochastic Gradient Descent algorithm implementation in RELION. The initial models were low-pass filtered to 20 Å and used for 3D auto-refinement to create primary fibril models with an initial twist of −1.15° (mouse fibril) or 1.54° (human fibril) and 4.8 Å (mouse fibril) or 4.7 Å (human fibril) helical rise, as evident from the cross-over distances and the layer line profiles of the 2D classes. The resulting reconstructions showed clearly separated β-sheets (*x*-*y* plane) and partially resolved β-strands along the fibril axis. The generated primary models indicated the presence of two identical protein stacks, which are related by a pseudo-2_1_ screw symmetry for both reconstructions (4.8/2 Å rise and (360°−1.15°)/2 twist for the murine fibril; 4.7/2 Å rise and (360° + 1.54°)/2 twist for the human fibril). Imposing this symmetry during reconstruction, in addition with *T* = 20, yielded a clear β-strand separation and side-chain densities. Three-dimensional classification with local optimization of helical twist and rise was used to further select particles in the murine dataset for a final high-resolution auto-refinement. The best 3D classes (3 out of 6) of the murine fibril were selected manually and reconstructed with local optimization of helical parameters using auto-refinement. For the human fibril dataset all particles were used for the final auto-refinement, without previous multi-class 3D classification. All 3D classification and auto-refine processes were carried out using a central part of 10% or 30% of the intermediate asymmetrical reconstruction^42^. The final reconstructions were post-processed with a soft-edge mask and an estimated map sharpening *B*-factor of −48 Å^2^ (murine fibril) and −84 Å^2^ (human fibril). The resolution was estimated from the Fourier shell correlation (FSC) at 0.143 between two independently refined half-maps.

### Model building and refinement

Both maps of murine and human fibril were sharpened by applying a B-factor of −50 Å^2^ using bfactor.exe (included with the FREALIGN distribution^43^). An initial poly-alanine model was built de novo by using Coot^44,45^. Known geometries of β-arches and arcades were considered for model building^46,47^. Once the backbone geometries were refined, side-chains were added. The clear densities for side-chains allowed us to unambiguously trace the orientation and register of the polypeptide chain. A protein stack consisting of six subunits was assembled and refined with PHENIX^48^ using phenix.real_space_refine^49^ (phenix-1.13–2998). Non-crystallographic symmetry (NCS) restraints and constraints were imposed on all chains using a high-resolution cutoff of 3.2 Å for the murine model and 2.7 Å for the human model. Initially, manually defined tight cross β-sheet restraints were imposed and were relaxed at the later stages of refinement. Steric clashes, Ramachandran and rotamer outliers were detected during refinement using Molprobity^50^ and iteratively corrected manually in Coot and refined in PHENIX. The protein stack was then fitted into the density of the opposing protein stack. The final dodecamer was first refined using rigid body refinement where each protein stack was defined as one rigid body and finally using global minimization and atomic displacement parameter (ADP) refinement with secondary structure and NCS restraints until convergence. B-factor (ADP) refinement yielded a final B-factor of 79.7 Å^2^ for the murine fibril model and 17.43 Å^2^ for the human fibril model^44^. Detailed refinement statistics are shown in Supplementary Table 3.

The final models were evaluated using Molprobity. For the murine model, no Ramachandran outliers were detected and 97.01% of the residues were in the favored region of the Ramachandran plot. Residues in the allowed region were residues Ala44 and Gly49, which are found in kink regions of the fibril protein. 98.08% of the residues of the human fibril model were in the favored region of the Ramachandran plot and no outliers were found.

An EMRinger score^51^ of 6.10 and 6.38, calculated for the murine and human dodecamer, respectively, highlights excellent model-to-map fit at high resolution and high accuracy of backbone conformation and rotamers. β-strands in the final model were analyzed using DSSP^52^ or STRIDE^53^ and defined manually.

### Image representation

Image representations of reconstructed densities and refined models were created with UCSF Chimera^54^. The following structures from the PDB were reproduced in the figures: hSAA1.1 (PDB 4IP8)^24^, human phosphoglycerate kinase (PDB 3C39)^55^.

### Reporting summary

Further information on experimental design is available in the Nature Research Reporting Summary linked to this article.

## Supplementary information

Supplementary Information

Reporting Summary

Source Data

## Data Availability

The reconstructed cryo-EM maps were deposited in the Electron Microscopy Data Bank with the accession codes EMD-8910 (murine) and EMD-9232 (human). The coordinates of the fitted atomic models were deposited in the Protein Data Bank under the accession codes 6DSO (murine) and 6MST (human). The source data underlying Figs. 2c and 6 and Supplementary Figs. 1, 2 and 3a are provided as a Source Data file. Other data that support the findings of this study are available from the corresponding authors upon reasonable request.
