# Supplementary material for: Cryo-EM fibril structures from systemic AA amyloidosis reveal the species complementarity of pathological amyloids
Source: Nat Commun. 2019 Mar 7;10:1104. doi: 10.1038/s41467-019-09033-z (PMC6405766; doi:10.1038/s41467-019-09033-z)
Supplement: Supplementary file 1 — Supplementary Information [file 41467_2019_9033_MOESM1_ESM.pdf]

Supplementary Information

Cryo-EM fibril structures from systemic AA amyloidosis reveal the species complementarity of pathological amyloids

F. Liberta, S. Loerch, M. Rennegarbe *et al*.

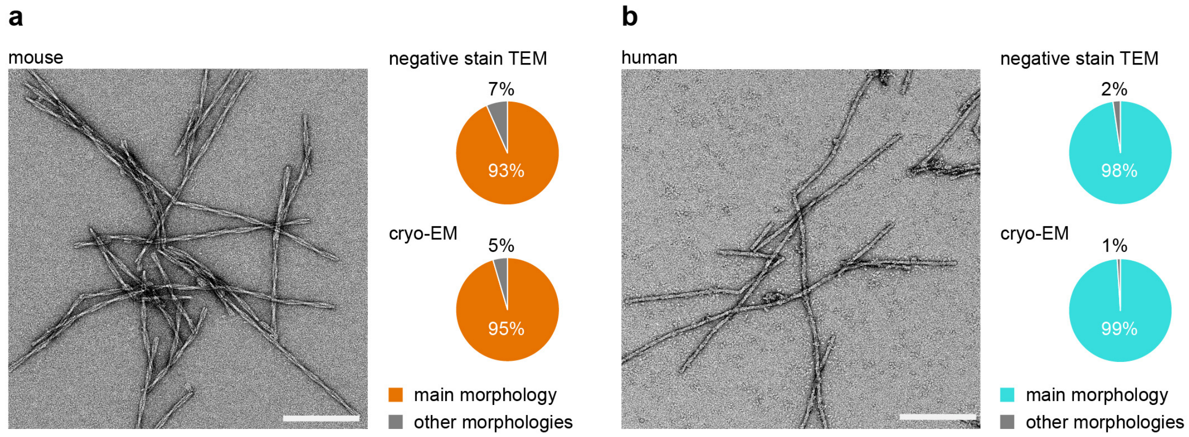

Supplementary Figure 1.

Morphological analysis of the extracted AA amyloid fibrils.

(a) Negative stain TEM image of murine AA amyloid fibrils. Quantification of the relative abundance of the main morphology as compared to other fibril morphologies based on negative stain TEM (n = 106 fibrils) and cryo-EM images (n = 220 fibrils). (b) Negative stain TEM image of human AA amyloid fibrils. Quantification of the relative abundance of the main morphology as compared to other fibril morphologies based on negative stain TEM (n = 150 fibrils) and cryo-EM images (n = 250 fibrils). Scale bars: 200 nm.

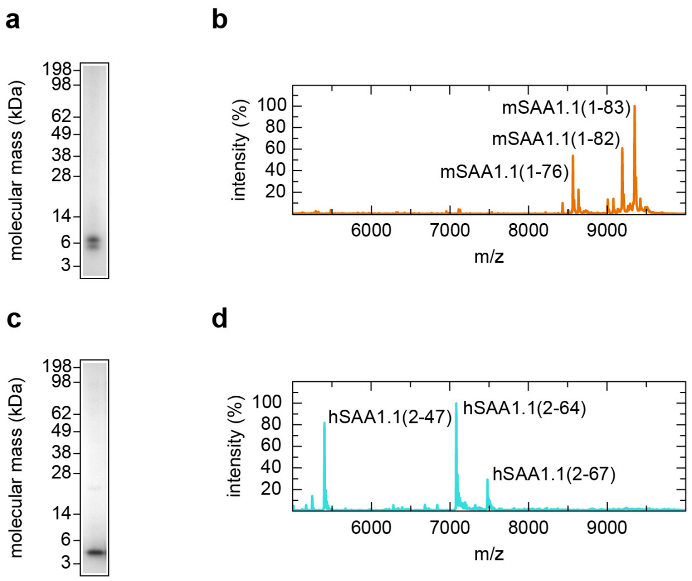

Supplementary Figure 2.

Molecular composition of the analyzed AA amyloid fibrils.

(a) Coomassie-stained denaturing protein gel prepared with murine AA amyloid fibrils. (b) Mass spectrum obtained with murine AA amyloid fibrils. (c) Coomassie-stained denaturing protein gel prepared with human AA amyloid fibrils. (d) Mass spectrum obtained with human AA amyloid fibrils.

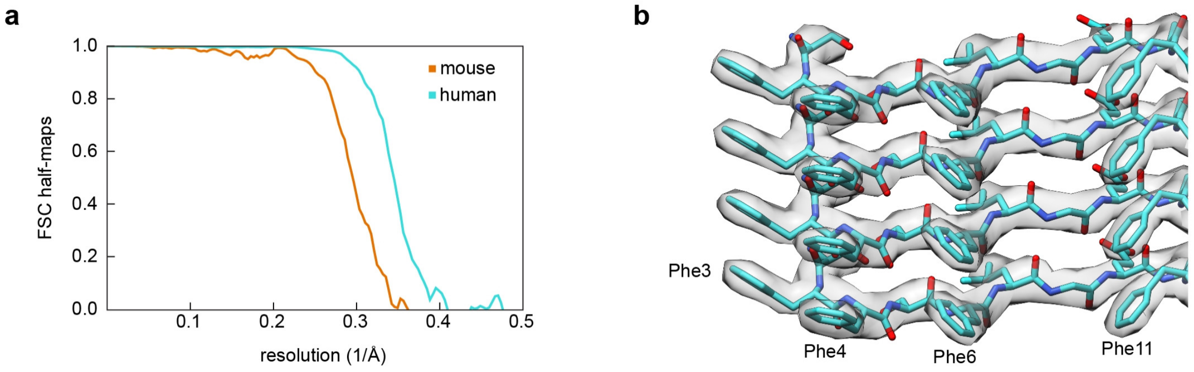

Supplementary Figure 3.

Resolution of the obtained 3D maps.

(a) FSC between the two half maps of the murine and human fibril. (b) Detail of the human fibril structure showing the density of specific side chains.

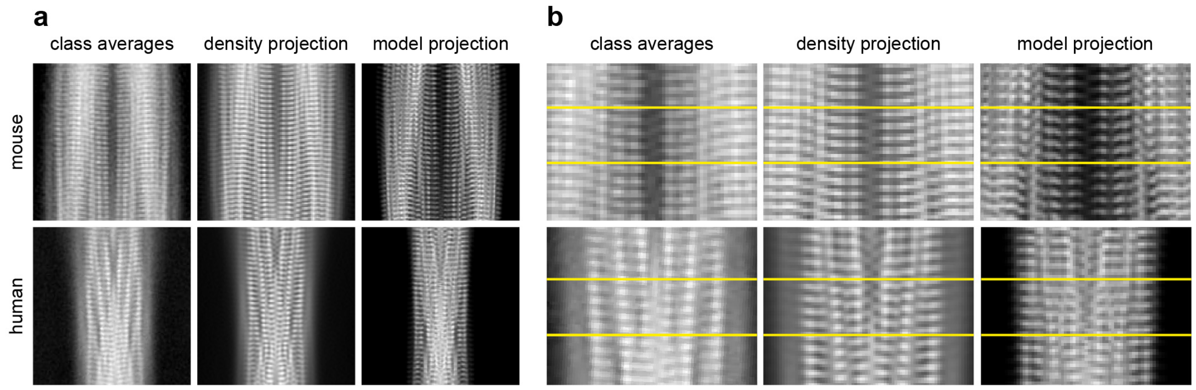

Supplementary Figure 4.

Comparison of the 2D averages with projections of the density and the model.

(a) Comparison of representative 2D class averages and corresponding 2D projections obtained with the density and the model of the murine and human fibril, respectively. (b) Close-ups of 2D class averages and corresponding 2D projections to highlight the stagger of the two protein stacks for the murine and human fibril, respectively. Yellow lines were drawn to guide the eye.

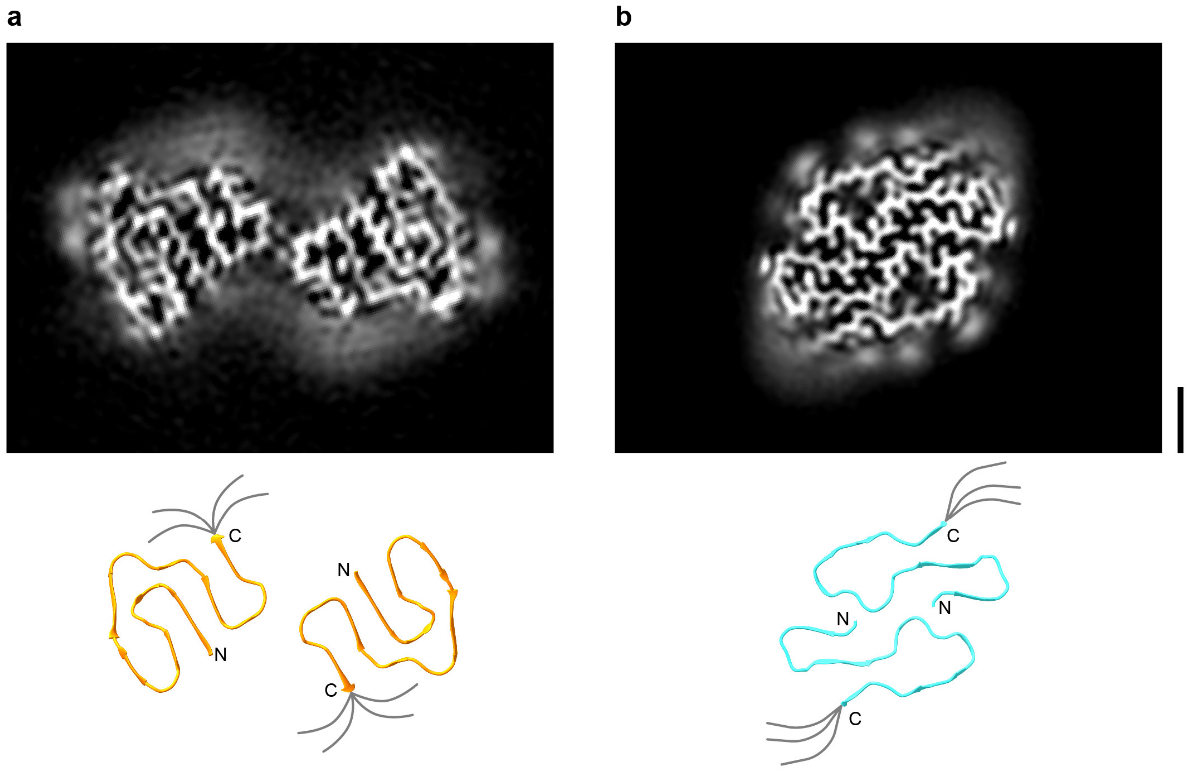

Supplementary Figure 5.

Ordered and disordered regions of the densities

(a) A 6.75 Å thick cross-sectional slice of the unmasked 3D map of the murine fibril. (b) A 6.24 Å thick cross-sectional slice of the unmasked 3D map of the human fibril (scale bar: 20 Å). Bottom: Ribbon diagrams showing one molecular layer of the murine and human fibril, respectively, with a schematic drawing of the disordered C-terminal tail (grey). Both fibril reconstructions show diffuse density outside the ordered fibril core. Some of the diffuse density corresponds to the possible location of the C-terminal tails of the fibril proteins. The remaining parts of the diffuse density are of uncertain origin and might represent a corona of molecules decorating the fibril core.

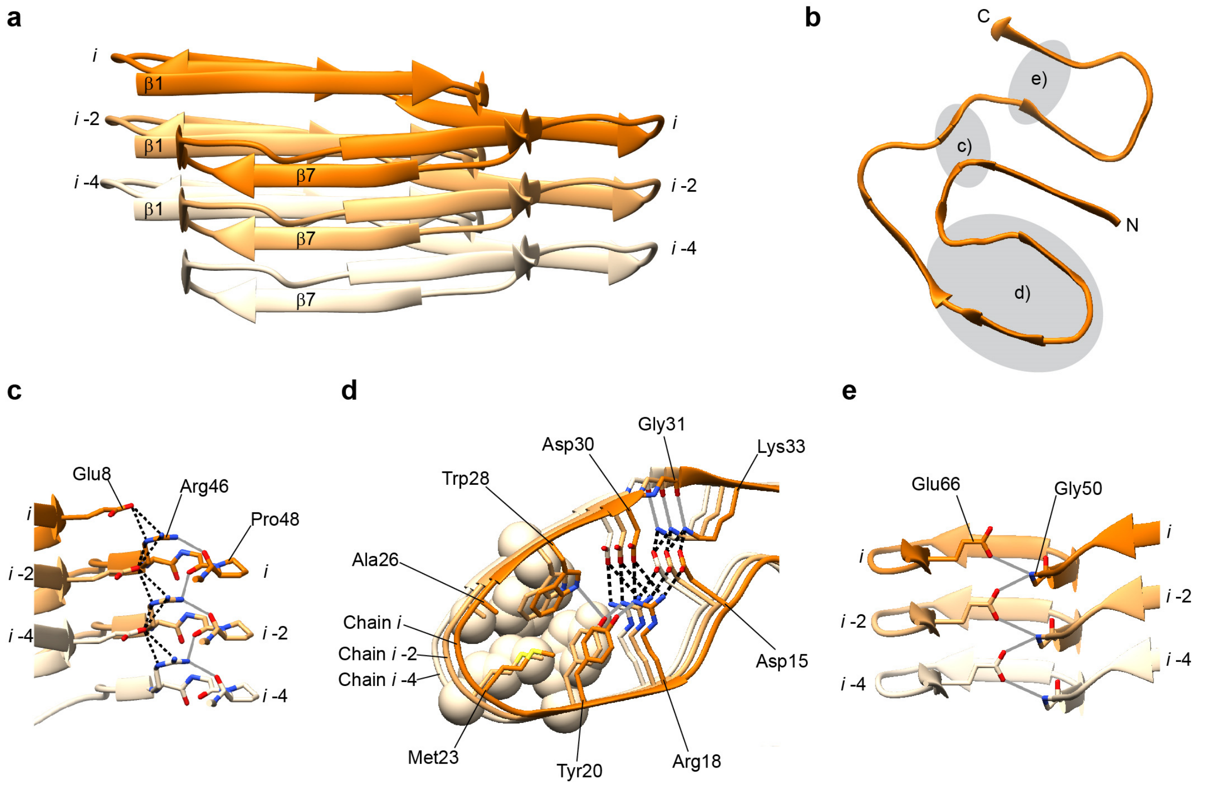

Supplementary Figure 6.

Buried ionic and polar interactions in the murine fibril.

(a) Side view of a fibril section demonstrating the intermolecular packing of strands β1 and β7. (b) Locations of the detail views shown in c-e annotated in a single peptide chain. (c) Arg46 (β6) forms electrostatic interactions with the carbonyl oxygen of Pro48 (β6-β7 arc), and intra- and intermolecular salt bridges with Glu8 (β1) in chains *i* and *i*-2. (d) Intramolecular salt-bridges are formed between Asp15, Arg18 (both β3), Asp30 (β4) and Lys33 (β5). Lys33 additionally forms an intermolecular salt-bridge with Asp15 of chains *i* and *i*-2. The boundary with the adjacent hydrophobic patch is formed by Tyr20 (β3) and Trp28 (β4), which form hydrogen bonds with Arg18 and with each other on one side, while packing with their respective hydrophobic surfaces against Met23 (β3-β4 arc) on the other side. (e) Glu66 (β9) forms bidentate electrostatic interactions with the backbone nitrogens of Gly50 of chains *i* and *i*+2. Salt bridges: black dashed; hydrogen bonds: grey.

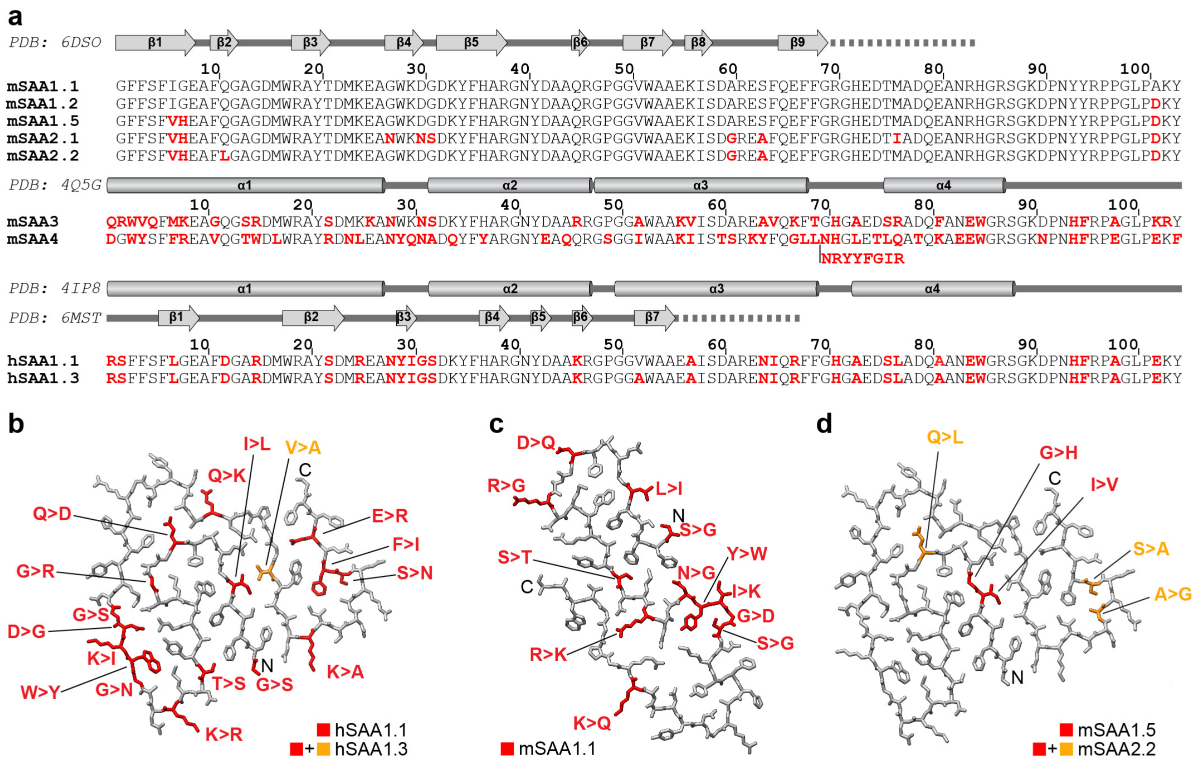

Supplementary Figure 7.

Comparison with naturally occurring SAA variants.

(a) Sequence comparison of different murine and human SAA proteins. Red: amino acid substitutions compared to mSAA1.1. Secondary structural assignments according to the respective PDB entries of the globular conformations of hSAA1.1[1] and mSAA3[2] and of the murine and human fibril protein are drawn above the respective precursor protein sequence. Cylinders: α-helices; arrows: β-strands. (b) The substitutions in hSAA1.1 and hSAA1.3 highlighted in the murine fibril structure. (c) The substitutions in mSAA1.1 highlighted in the human fibril structure. (d) The substitutions in mSAA1.5 and mSAA2.2 highlighted in the murine fibril structure.

Supplementary Table 1.

Molecular species detected by MS in mouse and human fibrils.

Peak position (m/z)	Assigned protein fragment	Theoretical mass (Da)
mouse fibrils		
8564.2	mSAA1.1(1-76)	8565.2
9194.4	mSAA1.1(1-82)	9193.8
9349.5	mSAA1.1(1-83)	9350.0
human fibrils		
5392.3	hSAA1.1(2-47)	5391.9
7074.1	hSAA1.1(2-64)	7073.7
7471.8	hSAA1.1(2-67)	7471.2

Left column: peak maximum position detected by MS; central column: assigned protein species; right column: calculated protein mass.

Supplementary Table 2.

Structural statistics of cryo-EM data collection and image processing.

Data Collection		
	mouse data set	***human data set***
Microscope	Titan Krios (Thermo Fisher Scientific)	Titan Krios (Thermo Fisher Scientific)
Camera	K2 Summit (Gatan)	K2 Summit (Gatan)
Acceleration voltage (kV)	300	300
Magnification	x 105,000	x 130,000
Defocus range (µm)	-1.3 to -5.5	-0.5 to -2.5
Dose rate (e^-/Å^2/s)	1.97 and 2.52	3.33
Number of movie frames	17 and 16	40
Exposure time (s)	10.2 and 8	12
Total electron dose (e^-/Å^2)	20	40
Pixel size (Å)	1.35	1.04
Reconstruction		
Box size (pixel)	210	270
Inter box distance (Å)	28	29
Number of extracted segments	137,956	93,025
Number of segments after 2D classification	93,045	91,872
Number of segments after 3D classification	21,024	-
Resolution, 0.143 FSC criterion (Å)	3.0	2.7
Map sharpening B-Factor (Å^2)	-48	-84
Helical rise (Å)	2.41	2.40
Helical twist (°)	179.44	180.79

Supplementary Table 3.

Structural statistics of model building and refinement.

Model composition		
	mouse	***human***
Non-hydrogen atoms	6588	5172
Number of chains	12	12
Model Refinement		
Resolution	3.3	2.8
Map CC (around atoms)	0.7836	0.8606
RMSD bonds (Å)	0.009	0.006
RMSD angles (˚)	0.928	0.800
All-atom clash score	1.53	1.43
Ramachandran outliers/favored (%)	0.00/97.01	0.00/98.08
Rotamer outliers	0.00	0.00
C-beta deviations	0	0
EMRinger score	6.10	6.38
EMRinger Zscore	13.86	13.07
Molprobity score	1.07	0.88

Supplementary References

1. Lu, J., Yu, Y., Zhu, I., Cheng, Y. & Sun, P. D. Structural mechanism of serum amyloid A-mediated inflammatory amyloidosis. *Proc. Natl. Acad. Sci. USA* **111,** 5189–5194 (2014).
2. Derebe, M. G. *et al.* Serum amyloid A is a retinol binding protein that transports retinol during bacterial infection. *eLife* **3,** e03206 (2014).
