# Supplementary material for: Cryo-EM fibril structures from systemic AA amyloidosis reveal the species complementarity of pathological amyloids
Source: Nat Commun. 2019 Mar 7;10:1104. doi: 10.1038/s41467-019-09033-z (PMC6405766; doi:10.1038/s41467-019-09033-z)
Supplement: Supplementary file 2 — Reporting Summary [file 41467_2019_9033_MOESM2_ESM.pdf]

nature research

Corresponding author(s): Marcus Fändrich and Matthias Schmidt

Reporting Summary

Nature Research wishes to improve the reproducibility of the work that we publish. This form provides structure for consistency and transparency in reporting. For further information on Nature Research policies, see Authors & Referees and the Editorial Policy Checklist.

Statistical parameters

When statistical analyses are reported, confirm that the following items are present in the relevant location (e.g. figure legend, table legend, main text, or Methods section).

n/a	Confirmed	
☐	☒	The exact sample size (*n*) for each experimental group/condition, given as a discrete number and unit of measurement
☐	☒	An indication of whether measurements were taken from distinct samples or whether the same sample was measured repeatedly
☒	☐	The statistical test(s) used AND whether they are one- or two-sided<br>*Only common tests should be described solely by name; describe more complex techniques in the Methods section.*
☒	☐	A description of all covariates tested
☒	☐	A description of any assumptions or corrections, such as tests of normality and adjustment for multiple comparisons
☐	☒	A full description of the statistics including central tendency (e.g. means) or other basic estimates (e.g. regression coefficient) AND variation (e.g. standard deviation) or associated estimates of uncertainty (e.g. confidence intervals)
☒	☐	For null hypothesis testing, the test statistic (e.g. *F*, *t*, *r*) with confidence intervals, effect sizes, degrees of freedom and *P* value noted<br>*Give P values as exact values whenever suitable.*
☒	☐	For Bayesian analysis, information on the choice of priors and Markov chain Monte Carlo settings
☒	☐	For hierarchical and complex designs, identification of the appropriate level for tests and full reporting of outcomes
☒	☐	Estimates of effect sizes (e.g. Cohen's *d*, Pearson's *r*), indicating how they were calculated
☒	☐	Clearly defined error bars<br>*State explicitly what error bars represent (e.g. SD, SE, CI)*

Our web collection on statistics for biologists may be useful.

Software and code

Policy information about availability of computer code

Data collection	SerialEM v3.5
Data analysis	RELION v2.1, MotionCor2 v1, Gctf v1.06, IMOD v.4.9.4, COOT v0.8.9, PHENIX v1.12 and v1.14, Fiji (ImageJ v.1.51n)

For manuscripts utilizing custom algorithms or software that are central to the research but not yet described in published literature, software must be made available to editors/reviewers upon request. We strongly encourage code deposition in a community repository (e.g. GitHub). See the Nature Research guidelines for submitting code & software for further information.

Data

Policy information about availability of data

All manuscripts must include a data availability statement. This statement should provide the following information, where applicable:

- Accession codes, unique identifiers, or web links for publicly available datasets
- A list of figures that have associated raw data
- A description of any restrictions on data availability

The reconstructed cryo-EM maps were deposited in the Electron Microscopy Data Bank with the accession codes EMD-8910 (murine) and EMD-9232 (human). The coordinates of the fitted atomic models were deposited in the Protein Data Bank under the accession codes 6DSO (murine) and 6MST (human).

April 2018

Field-specific reporting

Please select the best fit for your research. If you are not sure, read the appropriate sections before making your selection.

☒ Life sciences ☐ Behavioural & social sciences ☐ Ecological, evolutionary & environmental sciences

For a reference copy of the document with all sections, see nature.com/authors/policies/ReportingSummary-flat.pdf

Life sciences study design

All studies must disclose on these points even when the disclosure is negative.

Sample size	Murine: Fibrils were extracted from a single NMRI mouse. Human: Fibrils were extracted from a renal tissue sample of a single patient.
Data exclusions	No data were excluded from the analyses.
Replication	Electron microscopy data is based on a single mouse or single human patient, respectively. SDS-PAGE and mass spectrometry data were replicated successfully in at least four independent experiments. Representative data is shown.
Randomization	Not relevant to study. Single case Study.
Blinding	Not relevant to study. Single case Study.

Reporting for specific materials, systems and methods

Materials & experimental systems

n/a	Involved in the study	
☐	☒	Unique biological materials
☒	☐	Antibodies
☒	☐	Eukaryotic cell lines
☒	☐	Palaeontology
☐	☒	Animals and other organisms
☐	☒	Human research participants

Methods

n/a	Involved in the study	
☒	☐	ChIP-seq
☒	☐	Flow cytometry
☒	☐	MRI-based neuroimaging

Unique biological materials

Policy information about availability of materials

Obtaining unique materials	All unique materials used are readily available from the authors upon reasonable request or from standard commercial sources. Sources are presented in the methods section.

Animals and other organisms

Policy information about studies involving animals; ARRIVE guidelines recommended for reporting animal research

Laboratory animals	Female 6- to 8-week-old NMRI mice (Charles River Laboratories)
Wild animals	The study did not involve wild animals.
Field-collected samples	This study did not involve samples collected from the field.

Human research participants

Policy information about studies involving human research participants

Population characteristics	See Methods: Source of human AA amyloid fibrils; Age at death: 69; Gender: Female; Diagnosis: Chronic pulmonary obstructive disease, erosive seropositive rheumatoid arthritis, AA amyloidosis

Recruitment	Selected based on clinical findings at autopsy.
